# Toughening of Dental Composites with Thiourethane-Modified Filler Interfaces

**DOI:** 10.1038/s41598-019-39003-w

**Published:** 2019-02-19

**Authors:** Ana P. Fugolin, Daniel Sundfeld, Jack L. Ferracane, Carmem S. Pfeifer

**Affiliations:** 1 Department of Restorative Dentistry, Division of Biomaterials and Biomechanics, Oregon Health & Science University, Portland, OR USA; 2 UNINGA, Maringa University Center, in Maringa, PR Brazil

## Abstract

Stress of polymerization is one of the most significant drawbacks of dental resin composites, since it is related to poor marginal adaptation, postoperative pain, and secondary caries. Previous studies have shown that thiourethane oligomers incorporated into the organic matrix represents a promising strategy to reduce stress and increase fracture toughness in dental composites. However, this strategy promotes a significant increase of the viscosity system, which may represent a challenge for clinical application. The objective of this study was to functionalize the surface of inorganic filler particles with thiouretanes and evaluate the impact on mechanical properties and kinetics of polymerization. Our results showed that composites filled with thiourethane-silanized inorganic fillers showed up to 35% lower stress while doubling mechanical properties values. This was achieved with no prejudice to the viscosity of the material and following a clinically acceptable photoactivation protocol.

## Introduction

Resin composites are widely used for direct restorative procedures due to their esthetics and generally acceptable mechanical properties. However, composite restorations last an average of only about 10 years^1^, with failures being more commonly associated with material fracture and secondary decay^2^. Stress generation has been hypothesized to facilitate bacterial infiltration and biofilm formation at the interface between the tooth and the restoration, and when combined with composite material degradation by hydrolysis and enzymatic attack, may explain the relatively short life-time of composite restorations^3^. Therefore, research efforts have concentrated on modifying composition to render the composite material less prone to stress generation at the bonded interface^4^, and more resistant to fracture^5^.

Recent studies^6–9^ have demonstrated the potential of a relatively simple approach to improve conversion and fracture toughness of dental resin-based composites, while at the same time reducing polymerization stress. It has been shown that the addition of relatively small concentrations of thiourethane oligomers to the organic matrix of resin composites and luting cements leads to a 50–60% reduction in stress and a two-fold increase in fracture toughness^6^. Since the additive is simply incorporated into the traditional composite during formulation, no modification of the normal operatory technique is required, which in turn should facilitate its translation to clinical practice^6^. These benefits are achieved through the presence of pendant thiol functionalities on the backbone of the thiourethane additive^6^. As it has been widely demonstrated for thiol-ene^10^ and thiol methacrylate reactions^11^, thiols, via chain-transfer reactions, lead to delayed gelation and vitrification in vinyl-based polymer networks. This, in turn, allows for greater conversion to be achieved^12^, and for modulus development in the material to be delayed to higher conversion values^11^, ultimately leading to lower contraction stress generation. In addition, thiol-containing networks have been demonstrated to produce materials with narrow tan delta peaks in dynamic mechanical analysis^11^, characteristic of more homogeneous polymer networks^13^. This, combined with the flexibility of thio-carbamate bonds, results in increased toughness^8^ and reduced polymerization stress^14^.

One potential pitfall of including pre-polymerized additives, however, is the increase of viscosity, which limits the amount that can be incorporated into the monomer matrix. The addition of thiourethanes above 20 wt% in concentration increases the viscosity of the monomer mixture, which prevents the incorporation of adequate amounts of inorganic filler, and also leads to a slight decrease in elastic modulus^6^.

One possible way to incorporate the thiourethane oligomer in the composite material and overcome the viscosity issue is to attach it directly to the surface of the filler particle via common silanization procedures^15^. Others have demonstrated the use of polymer brushes to functionalize silicon-containing surfaces^16^ with reported decrease in polymerization stress, as well as evidence for strengthening mechanisms such as crack deflection^17^. Considering the average surface coverage with conventional methacrylate silanes (about 5%) for a composite with 70 wt% filler content, it should be possible to incorporate an equivalent amount of thiorethane in the composite. In that case, the oligomer is distributed throughout the material through attachment to the filler particles, with no negative effect on the viscosity of the monomer matrix itself.

The aim of the present study was to examine different properties of resin composites containing inorganic filler particles silanized with a thiourethane oligomer obtained by the combination of tri- and tetra-functional thiols with different isocyanates. The hypotheses of the study were: (1) Filler particles functionalized with thiourethane will be easily distributed into a resin matrix without negatively affecting kinetics of polymerization and depth of cure, and (2) fracture toughness will be improved and stress polymerization reduced in the groups with thiourethane-silanized particles.

## Results

### Thermogravimetric Analysis

The TGA results showed that the TU fillers had at least 48.5% more organic content than the commercially obtained methacrylate-silanized filler, i.e., particles functionalized with thiourethanes showed more oligomer available on the filler surface than the commercial particles silanized with methacrylate (Table 1).

**Table 1:** Percentage (%) of mass loss on the commercial methacrylate-silanized (SIL-MA), unsilanized and TU-silanized filler particles.

Groups	*% mass loss*
SIL-MA	3.67
UNS	1.47
TMP:BDI	5.56
TMP:HDDI	7.44
TMP:DHDI	5.45
PETMP:BDI	7.89
PETMP:HDDI	7.83
PETMP:DHDI	9.21

### Fracture toughness, polymerization stress, degree of conversion, and viscosity

Compared to the composite with the unsilanized filler and the control with a methacrylate silanized filler, the *K*_IC_ was significantly increased by the addition of thiourethane-silanized particles, and polymerization stress was significantly reduced (Table 2). In terms of *K*_*IC*_, the exception was TMP:BDI, which presented values statistically similar to SIL-MA control group. In terms of stress, all thiourethane-modified groups presented lower stress than the control, except for PETMP:BDI and PETMP:DHDI, which had similar values compared to the control. In general, the addition of thiourethane-silanized filler particles increased or did not influence the final DC and the viscosity of the composites. DC values ranged from 42–50% (Table 2). TMP:DHDI presented higher values than the controls (SIL-MA and UNS) and PETMP:BDI, and similar to TMP:BDI, TMP:HDDI, PETMP:HDDI, and PETMP:DHDI. Viscosity results ranged from 15.0–11.5 Pa.s and, in general, thiourethane-silanized filler groups presented similar or lower values than the controls.

**Table 2:** Means and standard deviations for fracture toughness (*K*_*IC*_, MPa m^1/2^), polymerization stress (MPa), degree of conversion (%), and viscosity (Pa.s) of all tested groups.

Groups	*K*_IC_	Stress	Degree of Conversion	Viscosity
SIL-MA	1.54 (0.11)^bc^	2.7 (0.1)^a^	44.0 (1.1)^b^	15.0 (0.8)^a^
UNS	0.83 (0.09)^d^	2.7 (0.2)^a^	42.1 (2.7)^b^	14.2 (0.5)^a^
TMP:BDI	1.36 (0.26)^c^	2.0 (0.2)^b^	50.4 (0.6)^ab^	14.1 (0.2)^ab^
TMP:HDDI	2.17 (0.16)^a^	1.8 (0.2)^b^	48.1 (1.1)^ab^	12.1 (0.2)^bc^
TMP:DHDI	1.94 (0.33)^ab^	1.8 (0.2)^b^	50.8 (1.1)^a^	14.8 (0.3)^a^
PETMP:BDI	1.99 (0.27)^a^	2.1 (0.3)^ab^	44.4 (1.0)^b^	13.6 (0.1)^ab^
PETMP:HDDI	2.02 (0.10)^a^	1.9 (0.3)^b^	49.2 (1.0)^ab^	11.5 (0.1)^c^
PETMP:DHDI	2.21 (0.28)^a^	2.3 (0.1)^ab^	48.6 (0.8)^ab^	14.3 (0.5)^a^
One-way ANOVA	p = 0.000	p = 0.000	p = 0.009	p ≤ 0.001

Values followed by the same superscript within the same test are statistically similar.

### Polymerization reaction kinetics

**Figure 1:**
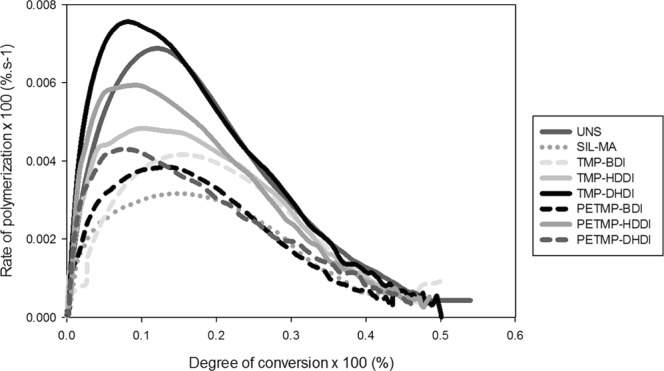
Degree of conversion as a function of the rate of polymerization for the control groups (SIL-MA and UNS) and thiourethane silanized fillers. Materials were photocured with 20 mW/cm^2^ for 300 seconds and the vinyl conversion was followed in real time.

There was statistical difference between the maximum rate of polymerization values (Rp max, Table 3). TMP-DHDI was greater than all groups, except for UNS and PETMP-HDDI. The lowest results were obtained for SIL-MA. Figure 1 shows the kinetic profiles for all tested groups. The degree of conversion at maximum rate of polymerization (DC at Rp max), used to estimate the conversion at the onset of vitrification, ranged from 7–14%, and showed a tendency for higher values with the groups containing BDI as the isocyanate, but which presented values similar to SIL-MA.

**Table 3:** Means and standard deviations for maximum polymerization rate (%·s^−1^) and degree of conversion at maximum polymerization rate.

Groups	Rp max(%·s^−1^)	DC at Rp max (%)
SIL-MA	3.26 (0.11)^c^	14.47 (1.97)^ab^
UNS	6.76 (1.41)^ab^	10.82 (0.95)^ab^
TMP:BDI	4.14 (0.39)^b^	14.66 (1.20)^a^
TMP:HDDI	4.88 (0.52)^b^	9.96 (0.62)^b^
TMP:DHDI	7.64 (1.42)^a^	10.45 (2.18)^ab^
PETMP:BDI	4.85 (0.27)^b^	14.40 (2.39)^ab^
PETMP:HDDI	6.73 (0.52)^ab^	7.37 (0.62)^b^
PETMP:DHDI	4.48 (1.94)^b^	7.22 (0.28)^b^
One-way ANOVA	p = 0.001	p = 0.000

Values followed by the same superscript within the same test are statistically similar (α = 0.05).

### Depth of polymerization

**Figure 2:**
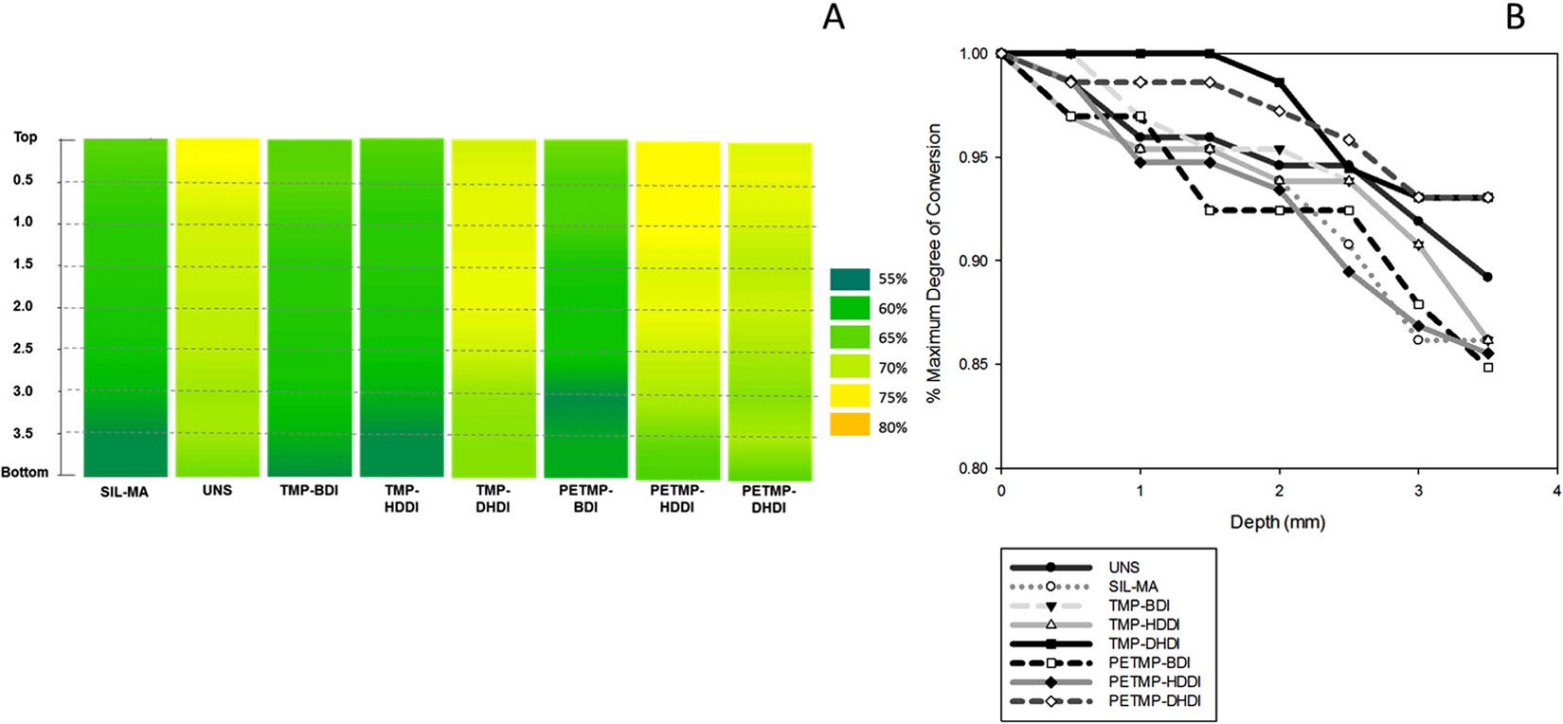
(**A**) Degree of conversion representation (%) according to different depths (mm) in 2D surface maps, and (**B**) Percentage of maximum degree of conversion (%) as a function of the depth (mm).

The degree of conversion as a function of depth is depicted in the 2-D maps shown in Fig. 2. Similar or higher degree of conversion was observed for TU silanized filler groups in relation to the control groups at all depths. In many cases, the depth of cure is defined as the depth at which degree of conversion (or hardness) falls below 80% of the top or maximum value. In this case, all materials showed a depth of cure of at least 3.5 mm, the full depth of these specimens.

## Discussion

The addition of thiourethane oligomers to methacrylate-based resin materials has been shown to increase the degree of conversion and fracture toughness, while simultaneously reducing polymerization stress^6–9^. In those previous studies, the limitation for the incorporation of oligomers (at about 20–30 wt% of the organic matrix mass) was dictated by an increase in viscosity, which in turn precluded the incorporation of inorganic fillers to the level necessary to produce adequate mechanical properties^6^. One way to possibly overcome this limitation was tested in this present study: to functionalize the thiourethane oligomers with trimethoxy silane functionality, so that the oligomer itself could be later tethered to the surface of the inorganic filler particle via common silanization procedures^15^. In this way, we aimed to improve the distribution of the oligomer within the composite, and to harness the same benefits obtained with the incorporation of the oligomer directly in the matrix, but without the drawback of significant increase of the viscosity. In fact, our results have shown that even for the fully formulated, highly filled composite, the viscosity average for the TU-silanized materials were similar or lower than the control groups (Table 2).

In the present study, the concentration of thiourethane used in the silanization procedure was kept constant at 2% of the total mass of the silanizing solution. The resulting fillers were characterized with thermogravimetric analysis to determine the extent of final surface coverage on the particles. It is important to note that even the unsilanized particles showed some mass loss after the test, likely due to the presence of organic contamination. The same contamination is unlikely to be present on the surface of the silanized filler particles, given the fact that the silanization process entails exposure to organic solvent in an acidic environment. In addition, the fillers were kept in sealed containers after silanization, so it is reasonable to assume that the mass loss in those cases was due to mainly the silane coating. The percent of surface functionalization based on the mass loss ranged from 5.45–9.21%, with the TU fillers showing at least 50% greater functionalization in comparison to the methacrylate control (Table 1). Since the filler particles in all formulations had the same size distribution, this difference in part likely reflects the higher molecular weight of the thiourethane silane (around 5 kDa^6^) as compared with the methacrylate silane (around 0.25 kDa), rather than indicating a more extensive surface coverage. Other studies evaluating silicon-containing surfaces functionalized with polymer brushes found similar results and attributed the mass loss to the formation of a coat of brush heap structures at the surface^16,18^. In the current study, given the loosely crosslinked nature of the oligomer formed, the surface coating is likely less organized than a conventional polymer brush^16^. In any case, when the percentage of filler surface coverage was calculated based on the mass loss from the TGA experiment, the average thiourethane concentration in the six composites containing the TU filler was found to be 7.2%. This is nearly one third of the amount of TU added to the resin matrix in previous studies to produce the same effects, such as increased fracture toughness and reduced polymerization stress^6^.

Degree of conversion for the TU filler composites (except for TMP-BDI) was on average 5–7% higher than for the control (SIL-MA). Previous studies have demonstrated the potential of the chain breaking events caused by the presence of thiols to delay gelation and vitrification in radical polymerizations^19^. In this reaction, the chain-transfer events lead to a delay of the point in conversion at which monomer mobility limitations hamper polymerization and, thus, allow higher final conversion and delayed modulus development, ultimately delaying and reducing stress development^20^. Based on these mechanisms and our previous results obtained when incorporating the thiourethane oligomers directly into the organic matrix, it was expected that thiourethane-silanized materials would not only show increased degree of conversion, but that they also would be able to reduce the rate of polymerization and delay vitrification^7^. The delay in vitrification was estimated by the conversion registered at the onset of deceleration (Rp_max_). Contrary to what was expected for the reaction rate, all of the groups containing the thiourethane-silanized filler showed equivalent or higher maximum rate of polymerization compared to the methacrylate control. However, all of the composites with TU filler showed equivalent or significantly lower DC at Rp_max_. In any case, there was no significant correlation between the measured stress and the Rp_max_ or measured stress and DC at Rp_max_. One possible explanation is that the overall concentration of thiourethane in the present study was lower, and therefore, the effects on network formation were not as marked as had been seen previously. In addition, because the incorporation of the thiourethanes to the filler surface did not affect the material’s viscosity, its effect on early mobility restrictions observed with the thiourethanes added directly into the matrix was not observed here. Even though gelation and vitrification were not directly assessed in this study, the kinetic profiles are strong indicators for those events^21^, and it can be speculated that these did not play a role in the decreased stress observed, which then must be explained by other factors.

**Figure 3:**
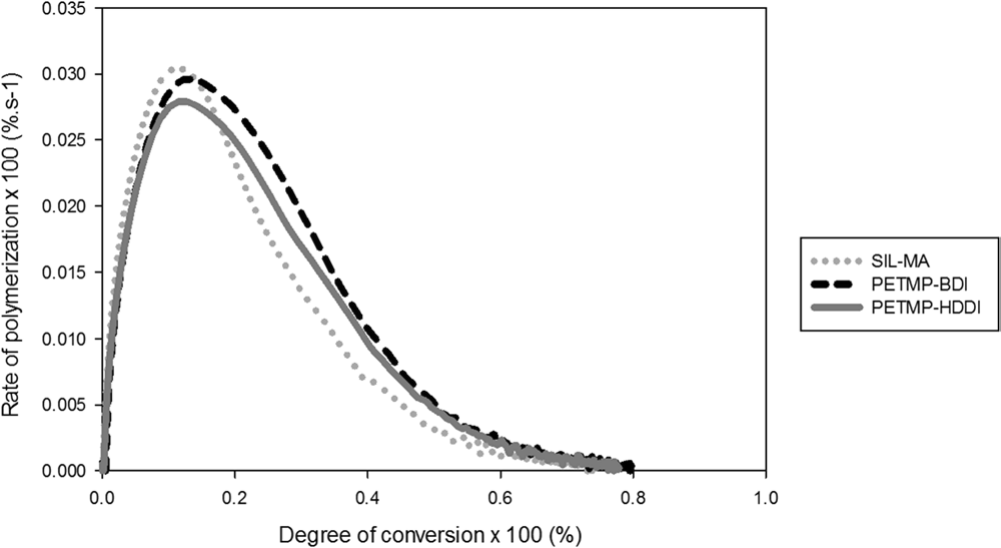
Degree of conversion as a function of the rate of polymerization for the methacrylate-silanized control group and thiourethane-silanized fillers groups. Materials were photocured with 650 mW/cm^2^ for 300 seconds and the vinyl conversion was followed in real time.

A specifically low irradiance was used for the kinetics test in order to highlight the differences between the tested thiourethanes. However, to better reproduce clinically relevant conditions, selected groups (Sil-MA, PETMP:BDI and PETMP:HDDI) were evaluated for polymerization kinetics under the same conditions described previously but at a much higher irradiance of 650 mW/cm^2^. The results (Fig. 3) showed that the polymerization was much faster than with the low irradiance, as expected, but also that there was no significant difference between the groups in terms of Rp_max_ (3.04, 2.97 and 2.88%.s^−1^, respectively) and DC at Rp_max_ (11.8, 13.1 and 13.2%, respectively). Further, the TU-silanized groups showed 4–6% higher final DC than the methacrylate-silanized control, which is in agreement with our previous results^22^. In combination with the depth of cure data, this shows the general ability of the thiourethanes to maintain or increase the degree of conversion and improve homogeneity of the polymer network, likely due to the chain-transfer mechanism leading to the delayed gelation/vitrification, thus maximizing the degree of conversion before the reaction becomes diffusion-controlled^11^. Among the tested thiourethanes, PETMP–containing materials showed higher conversion than the control at the bottom of the specimen for two out of the three groups, whereas TMP only led to higher conversion at the bottom for the combination with the DHDI isocyanate, as shown in the heat map plots. This is at least in part explained by the fact that PETMP is tetrafunctional and TMP trifunctional, which results in higher concentration of thiol functionalities pendant from the thiourethane backbone^6^ available on the filler surface to undergo the chain-transfer mechanism. The cyclic isocyanate DHDI led to consistently high and more uniform conversion throughout the specimen, likely because it represented an intermediate between the stiff backbone imparted by the presence of the aromatic BDI and the highly flexible backbone imparted by HDDI. Interestingly, for the more highly crosslinked PETMP-containing TUs, the use of HDDI led to higher initial DC and then essentially equivalent DC as with DHDI throughout the specimen^9,23^. As the refractive index is expected to increase with the addition of any of the TU silanes, leading to improved light transmission^22^, the depth of cure was expected to increase for all TU-containing groups. However, as shown on the plot for percent cure in depth in relation to the conversion at the top, two of the three PETMP are actually the lowest depth of cure in percentage, but when PETMP is paired with DHDI the depth of cure is still high. In fact, the DHDI-containing groups present the highest values in terms of cure throughout, and at the greatest depth (though not the highest absolute conversion value at the top). The TMP groups present the best percent depth of cure with DHDI, and then even slightly better than PETMP when paired with BDI and HDDI. It is important to highlight that the degree of conversion is high throughout the specimens, so these differences are likely non-clinically relevant. In summary, this study demonstrates that TU-additives are capable of maintaining the high conversion at the top throughout the specimen much more efficiently than the non-modified control, and highlights the influence of the TU structure on depth of cure.

There was a significant 15–35% reduction in polymerization stress for materials containing thiourethane silanized filler particles. As previously mentioned, this was true in spite of the apparent lack of delayed gelation/vitrification phenomena, at least as inferred from the polymerization kinetics curves. In this case, other factors likely contributed to the reduced stress values observed. The TGA results demonstrate that the filler coverage with the thiourethane silane represents between 5.4 to 9.2% of the mass of the filler, as compared to only 3% for the methacrylate silane controls. Two factors need to be considered: 1. the same amounts by weight of each molecule were used during the silanization, and 2. each methacrylate silane molecule presents only one trimethoxy silane functionality, compared to multiple functionalities for the TU oligomer silane. Therefore, it is possible that the methacrylate silane formed a mono-layer of molecules, and the TU-silane likely formed a much thicker layer on the filler surface. Also, due to the greater complexity of the molecular structure of the TU-silane, even with a greater mass of silane, it is very unlikely that the surface was covered to the same extent as with the methacrylate silane. It follows that the stress reduction mechanism may stem from two factors. The first is the possibility for the surface of the particle to be more exposed (“unsilanized”) for the TU silane than for the methacrylate controls, which would then reduce stress because the filler particle in part actually acts as a void. This type of behavior was shown when non-silanated nanoparticles were used to fill dental composites^24^. However, this was not observed here, as the non-silane treated filler showed equivalent stress to the methacrylate-silane control. This suggests that the fillers acting as pores is not likely the explanation for this result. The second, and arguably the most relevant factor relates to the flexible and tough nature of the thiourethane networks, as extensively demonstrated in the literature^7,9,23,25^. It can be speculated that the presence of the TU around the filler particles acted to locally relieve the stress developed during polymerization, which then resulted in overall less stress generation in the composite. In fact, this mechanism has been proposed in other studies evaluating the efficacy of polymer brush functionalization on stress reduction at interfaces with silicon-containing materials^17^. Polymer brushes or tethered polymers may stretch away from the surface and accommodate for the change in free volume that accompanies polymerization^26,27^. Another possible explanation is active strand behavior, demonstrated recently for urethane-containing materials^28^.

It is important to note that the stress reduction (and also the effect on polymerization kinetics) were dependent on the molecular structure of the precursors used to synthesize the thiourethane silanes. Overall, the groups where the tri-functional thiol (TMP) was used as a precursor exhibited lower stress than groups containing the tetra-functional thiol (PETMP), which then correlates with the likely greater degree of crosslinking provided by PETMP. This has been previously shown in studies using dynamic mechanical analysis to evaluate crosslinking (via the rubbery plateau method)^29^. Therefore, the more crosslinked material likely produced a stiffer network around the filler particle, which was then less able to relieve stress. Interestingly, the use of more rigid isocyanate precursors (BDI, containing aromatic moieties) did not seem to influence stress development more or less than the other TU silanes, suggesting that the structure of the TU oligomer itself, independent of its base monomer structure, had the greatest influence on stress reduction. The loss and storage modulus obtained with dynamic mechanical analysis (data shown in the Supplementary Information) also demonstrate the lack of correlation between the TU structure and property development, corroborating the fact that the thiocarbamate-based structure has a greater influence on stress development and mechanical reinforcement.

The same mechanisms used to explain stress relief and ultimately overall stress reduction can be used to explain the fracture toughness results. Thiourethane silanized particle groups showed a 25–85% increase in fracture toughness compared to the control groups. Fracture toughness may be important to help predict the clinical behavior of the dental composite restoration as it is related to chipping and bulk fracture^30,31^. When thiourethanes are added into the methacrylate matrix, the improvement of the mechanical properties is attributed to the flexible nature of the covalent bonds formed between the thiol on the thiourethane with the methacrylate via chain-transfer reactions^8^, leading to a more uniform and tougher network^14,32^. In the present study, those flexible bonds were formed around the filler particles, a region where stress concentration occurs^33^. This, in conjunction with the tough nature of the thio-carbamate bonds in the oligomer structure, likely led to some crack deflection or crack shielding mechanism around the particles^33,34^, as has been suggested by nanomechanical analysis of silicon interfaces treated with polymer brushes. It is also possible that the thiourethane-based silane bonding to the methacrylated matrix was more efficient due to its multiple methoxysilane functionalities, which could have enhanced the filler particle-matrix interaction leading to an increase in mechanical properties. Furthermore, fracture toughness is correlated to tensile stresses build up around the filler particles, and the efficiency of silane treatment plays an important role in gap formation^35^. Therefore, it can be speculated that the interfacial bonding between the organic matrix and filler particles was strong enough to resist the polymerization shrinkage, creating fewer gaps. PETMP groups showed better performance than TMP (increase between 40–85% for PETMP groups *versus* 0–40% for TMP groups) which may be due to PETMP being tetrafunctional while TMP is trifunctional. The greater crosslinking improved the mechanical strength of the matrix around the particles, and while that likely resulted in less efficient stress relief, as described earlier, it led to reinforcement of the material overall. The isocyanate structure did not influence the results, though a trend could be observed for increased mechanical properties and decreased polymerization stress, which could be due to the more flexible nature of this material^9,23^.

In general, the results of this study demonstrate that the incorporation of thiourethane silanized fillers into resin composites led to increased conversion at faster rates than the control, while increasing fracture toughness, and reducing stress of polymerization. Even though the microbiological aspect was not considered on the scope of this investigation, these materials might be able to reduce gap formation, and hamper bacterial colonization at the bonded interface, ultimately helping avoid restoration failures due to secondary decay. Studies are underway utilizing a secondary caries model to test this hypothesis.

## Materials and Methods

### Material Composition

The experimental composites were composed of Bis-phenol A diglycidyl dimethacrylate (Bis-GMA), urethane dimethacrylate (UDMA) and triethylene glycol dimethacrylate (TEGDMA) at a 50:30:20 mass ratio. All monomers were purchased from Esstech (Essington, PA, USA). Photoinitiators were added to the monomers as follows: 0.2 wt.% of dl-camphoroquinone (Polysciences Inc., Warrington, PA, USA), 0.8 wt.% of a tertiary amine (EDMAB – ethyl 4-dimethylaminobenzoate; Avocado, Heysham, England), and 0.2 wt.% inhibitor (BHT – 2,6-di-tert-butyl-4-methylphenol; Sigma–Aldrich, St. Louis, MO, USA).

Six thiourethane oligomers were synthesized in solution in the presence of catalytic amounts of triethylamine. Two multi-functional thiols – pentaerythritol tetra-3-mercaptopropionate (PETMP) or trimethylol-tris-3-mercaptopropionate (TMP) – were combined with three di-functional isocyanates – 1,6-hexanediol-diissocyante (HDDI) or 1,3-bis(1-isocyanato-1-methylethyl) benzene (BDI) (aromatic) or 1-isocyanato-4-[(4-isocyanatocyclohexyl) methyl] cyclohexane (DHDI) in 60 ml of methylene chloride. In addition, 1 mol of 3-(triethoxysilyl)propyl isocyanate was also added to each of the six combinations described above – this is the source of trimethoxy silane to be used for the subsequent silanization step. The reaction was catalyzed by triethylamine. The isocyanate:thiol mol ratio was kept at 1:2.5 (with thiol in excess) to avoid macro-gelation of the oligomer during reaction, according to the Flory–Stockmayer theory^21^, leaving pendant thiols and trimethoxy silanes. Oligomers were purified by precipitation in hexanes and rotoevaporation, and then characterized by mid-IR and NMR spectroscopy^6^. The disappearance of the isocyanate mid-IR peak at 2270 cm^−1^ and the appearance of NMR resonance signals at 3.70 ppm were used as evidence for completion of isocyanate reaction and thiourethane bond formation, respectively^36^.

For the silanization procedures, thiourethane oligomers were combined with 65 ml of an ethanol: distilled water solution (80:20 vol%), previously acidified by the addition of glacial acetic acid (pH = 4.5). Thiourethane was added at 2 wt%, in relation to the solution mass. Five grams of neat barium silicate glass filler (average size = 1.0 μm; Kavo Kerr Corporation, Orange, CA) was added to the solution, kept under magnetic stirring for 24 hours, filtered, and dried for 4 days in an oven at 37 °C.

The TU fillers were introduced at 50 wt% to the monomer matrix with a centrifugal mixer (DAC 150 Speed Mixer, Flacktek, Landrum, SC, USA) operated for 2 min at 1800 rpm. All procedures were carried out under yellow light.

Control groups were prepared with a commercially available unsilanized (UNS) and methacrylate-silanized (SIL-MA) barium glass filler particles (average size = 1.0 μm; Kerr Corporation, Orange, CA). All photocuring procedures were carried out using a mercury arc lamp (EXFO Acticure 4000 UV Cure; Mississauga, Ontario, Canada) filtered at 320–500 nm (light guide diameter = 5 mm). In order to verify the achieved functionalization and its efficiency, the different filler particles were analyzed by thermogravimetric analysis (TGA) over a temperature range of 50 °C to 850 °C at 10 °C/minute.

### Fracture Toughness (*K*_IC_)

*K*_IC_ was determined using the single-edge notch beam method. Specimens were fabricated according to ASTM Standard E399–90^37^ by filling a 5 × 2.5 × 25 mm (n = 6) split steel mold with a razor blade insert providing a 2.5 mm long notch in the middle of the specimen. The resin composites were photoactivated for 2 min on each side at an irradiance of 800 mW/cm^2^. The light guide was kept 7 cm away from the sample and a radiometer (Power Max 5200 – Laser Power Meter, Molectron, Portland, OR, US) was used to measure the power output from the mercury arc lamp and guarantee that 800 mW/cm^2^ irradiance reached the top surface of the sample. The bending fracture test was performed using a universal test machine (MTS Criterion, Eden Prairie, MN USA) at a cross-head speed of 0.5 mm min^−1^ and *K*_IC_ was calculated according to the following equation (1):

1 $$Kic=\frac{3PL}{2B{W}^{\frac{3}{2}}}\{1.93{(\frac{a}{W})}^{\frac{1}{2}}-3.07{(\frac{a}{W})}^{\frac{3}{2}}+14.53{(\frac{a}{W})}^{\frac{5}{2}}-25.11{(\frac{a}{W})}^{\frac{7}{2}}+25.8{(\frac{a}{W})}^{\frac{9}{2}}\}$$

where *P* is load at fracture (N), *L* is the length, *W* is the width, *B* is the thickness, and *a* is the notch length (all in mm).

### Polymerization Stress

Polymerization stress in real-time was analyzed using a cantilever beam apparatus (Bioman) previously described^38^. The surface of the fused silica plate was treated with a thin layer of silane ceramic primer (3 M ESPE, St. Paul, MN, USA) and the surface of the 5 mm diameter steel piston with Z-Prime Plus (Bisco Inc., IL, USA). The composite was then inserted into the 1-mm gap between the upper rod and the lower glass slide and shaped into a cylinder, removing the excess. The samples were light-cured through the glass using the light curing unit for 40 s (n = 5) at 280 mW/cm^2^. Data were recorded for 10 min on a computer and the final shrinkage-stress calculated.

### Kinetics and Degree of Conversion

Three specimens (8 mm in diameter and 0.6 mm in thickness), laminated between two glass slides, were cured and then measured with near-infrared (NIR) spectroscopy (Nicolet 6700, Thermo Electron Corporation, Waltham, MA, US) to calculate the degree of conversion (DC). The area corresponding to the methacrylate double bond overtone at 6165 cm^−1^ ^39^ was recorded before and after 300 s of irradiation with the light source located 4 cm from the surface of the glass slide, delivering 20 mW/cm^2^ directly to the specimen. Real-time monitoring of the polymerization kinetics for 300 s was carried out on specimens of the same size at 2 scans per spectrum with 4 cm^−1^ resolution.

### Depth of polymerization – 2D Mapping with IR-Microscopy

Samples (2 × 5 mm cross-section and 5 mm depth) were prepared using silicon molds (n = 3) and photocured through a glass slide for 300 s at 20 mW/cm^2^. After 7 days dry storage, the samples were embedded in slow curing epoxy resin and then sectioned using a diamond saw (Accutom-50) to obtain two slices of 0.4 mm thickness. The degree of conversion of the vinyl groups with depth was measured in near-IR (6165 cm^−1^) using an IR microscope attached to a spectrometer bench (Continuum and 6700, Thermo Fisher). 2D surface maps of conversion were set up with 200 µm step sizes.

### Viscosity

Viscosity was measured in a cone-plate rheometer (ARES, TA Instruments, New Castle, DE, USA). 1.03 g of each unpolymerized resin was placed between 20-mm diameter plates and tested at 1 Hz with a gap size of 0.3 mm (n = 3).

### Statistical Analysis

Data for each property was tested for normality and homocedasticity (Anderson-Darling and Levene tests, respectively), and then analyzed with one-way ANOVA and Tukey’s test for multiple comparisons. The significance level was set at 95%.

## Supplementary information

Supplementary Information - Dynamic Mechanical Analysis

## Data Availability

The data generated and analyzed during the current study are available from the corresponding author on reasonable request.
