# Supplementary material for: Toughening of Dental Composites with Thiourethane-Modified Filler Interfaces
Source: Sci Rep. 2019 Feb 19;9:2286. doi: 10.1038/s41598-019-39003-w (PMC6381174; doi:10.1038/s41598-019-39003-w)
Supplement: Supplementary file 1 — Supplementary Information - Dynamic Mechanical Analysis [file 41598_2019_39003_MOESM1_ESM.docx]

**Supplementary Information**

**TOUGHENING OF DENTAL COMPOSITES WITH THIOURETHANE-MODIFIED FILLER INTERFACES**

*Ana P. Fugolin; Daniel Sundfeld; Jack L. Ferracane; Carmem S. Pfeifer**

**Materials and Method**

***Dynamic Mechanical Analysis***

Dynamic mechanical analysis (DMA) was performed in the temperature range of −50 °C to 280 °C with a heating rate of 3°C per min in tension mode (sinusoidal stress at 1 Hz). Five samples (15 mm × 3 mm × 1 mm) were irradiated for 2 min on each side at an irradiance of 800 mW/cm^2^ using a mercury arc lamp (EXFO Acticure 4000 UV Cure; Mississauga, Ontario, Canada) filtered at 320-500 nm (light guide diameter = 5 mm). The bars were post-cured at 180°C for 24 hours until conversion exceeded 98%.

**Results and Discussion**

Dynamic mechanical analysis was used here to gain insight into the polymer network formed in the presence of thiourethane coatings on the surface of inorganic fillers used in composites. The storage modulus (E’) relates to the mechanical energy stored by the material during a loading cycle, and corresponds to the shape recovery ability and stiffness of the polymer. The loss modulus (E”) indicates the ability of the polymer to disperse mechanical energy through internal molecular motions. Results are shown in Figure S1. In summary, the surface filler functionalization did not affect E’ nor E”, regardless of the filler content, demonstrating that the stress reduction observed in this study did not come at the expense of bulk mechanical properties, but instead, can be credited to stress-relieving chain-transfer reactions leading to delayed gelation/vitrification. In addition, this result corroborates the argument that the increase in fracture toughness seen in the thiourethane-functionalized filler groups is related to the more homogeneous and tougher polymer networks afforded by the presence of pending thiols and thiol-carbamate bonds, respectively ^8,14^, right at the surface of the filler. There is no indication, therefore, that the slight differences in coating mass observed with TGA influenced the fracture toughness results and, instead, the increase in the resistance to crack propagation was due to the presence of the thiourethane oligomer right where stress concentrates the most.

**
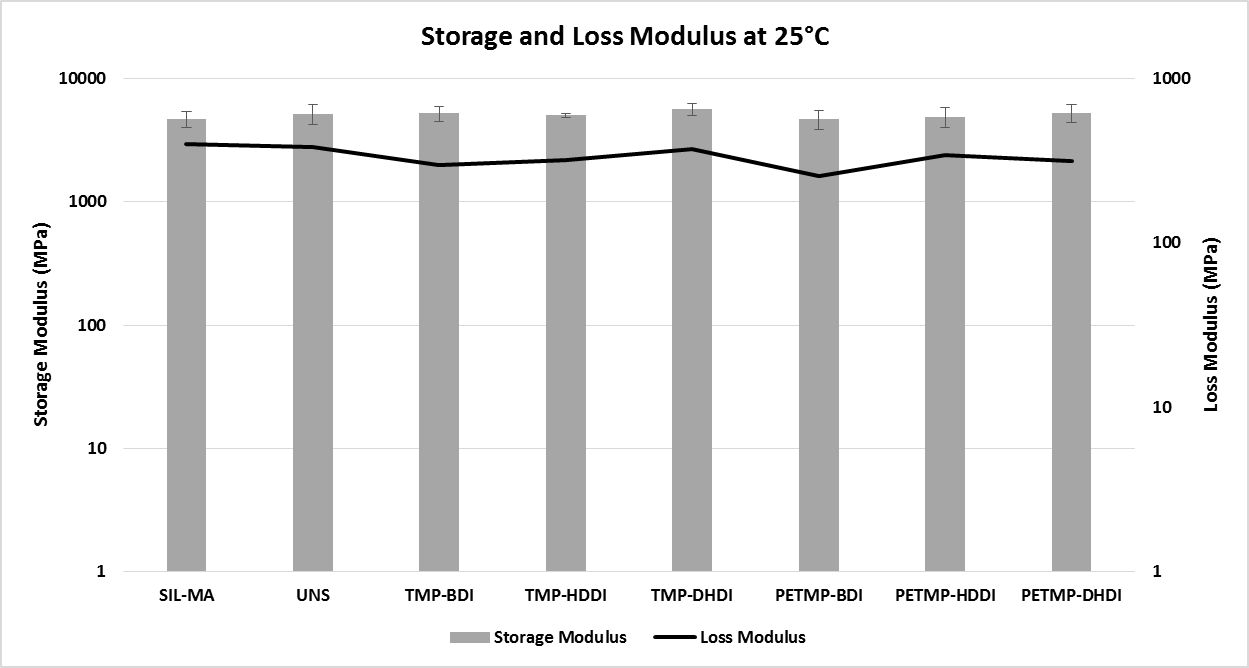
**

**Figure S1:** Storage (E’) and Loss Modulus (E”) for resin composites filled with 50 wt% filler particles functionalized at 25°C at 1 Hz.
